# Recovering wasted nutrients from shrimp farming through the combined culture of polychaetes and halophytes

**DOI:** 10.1038/s41598-021-85922-y

**Published:** 2021-03-23

**Authors:** Daniel Jerónimo, Ana Isabel Lillebø, Javier Cremades, Paulo Cartaxana, Ricardo Calado

**Affiliations:** 1 ECOMARE and CESAM and Departamento de Biologia, Universidade de Aveiro, Campus Universitário de Santiago, 3810-193 Aveiro, Portugal; 2 Coastal Biology Research Group (BioCost), Facultad de Ciencias and CICA, Universidade da Coruña, 15071 A Coruña, Spain

**Keywords:** Marine biology, Environmental biotechnology, Field trials, Sustainability

## Abstract

The bioremediation and biomass production of organic extractive organisms (polychaetes *Arenicola marina*, *Hediste diversicolor* and halophyte *Salicornia ramosissima*) was assessed in an integrated multi-trophic aquaculture (IMTA) framework. Culture trials were performed outdoors using the nutient rich effluent from a shrimp farm employing recirculated aquaculture systems. Similar bioremediation efficiencies were obtained in cultures using a single polyculture tank (1 T) or two trophic levels separated tanks (2 T; ≈ 0.3 and 0.6 m^2^ operational area, respectively), with a reduction of 74–87% for particulate organic matter (POM), 56–64% for dissolved inorganic nitrogen (DIN) and 60–65% for dissolved inorganic phosphorus (DIP). *Hediste diversicolor* adapted well to culture conditions, reaching densities up to 5.000 ind. m^−2^ (≈ 78–98 g m^−2^). *Arenicola marina* failed to cope with water temperature that exceeded the species thermal limits, displaying a survival < 10% (20 °C often pointed as the maximum thermal threshold for this species). Productivity of *S. ramosissima* with 1 T was about twice that obtained with 2 T (≈ 150–170 and ≈ 60–90 g FW m^−2^ edible aboveground biomass, respectively). The yellowish coloration of cultured plants was likely due to the chemical oxidation and rapid sand filtration pre-treatment applied to the brackish groundwater used in the aquaculture facility, that removed iron (and probably other essential elements). Overall, 1 T design combining *H. diversicolor* and *S. ramosissima* displayed the best bioremediation performance and biomass production, while also allowing reducing in half the operational area required to implement this IMTA framework.

## Introduction

Integrated multi-trophic aquaculture (IMTA) is an ecosystem-based approach where species of different trophic levels are integrated to maximize the recovery of nutrients introduced in the production system^1–3^. In this context, unused nutrients (uneaten feed and faeces) derived from fed species (e.g. fish and crustaceans) that are commonly wasted through farm effluents are recovered into valuable extractive species biomass, with effluent bioremediation also being achieved^1–3^. Saltwater aquaculture (marine and brackish-water) is paramount to fulfil dietary needs, contributing to food security worldwide^4^. In 2018 this sector represented approximately 56% and 46% of the volume and value generated by aquaculture in total (global values above 114 million tonnes and USD 263 billions)^5^. The vast majority of farmed fish and crustaceans (≈ 20% and 58% of the volume and value of saltwater aquaculture production) are carnivorous species which continue to require feeds that contain fish meal and fish oil in their composition, two increasingly scarce resources^5,6^. These raw materials, still continue to be considered the most nutritious and most digestible ingredients for major saltwater farmed species, as well as major sources of essential long-chain omega-3 fatty acids (e.g., eicosapentaenoic acid [EPA] and docosahexaenoic acid [DHA])^5^. Saltwater farmed species are known to excrete between 50 to 80% of feed nitrogen (N) and 35 to 85% of feed phosphorus (P), which represents an economic constrain, as farm effluents need to be treated to meet legal regulations to enable their release into the aquatic environment^7^. This constrain can be overcome if such excess of nutrients are extracted, e.g., through bioremediation, from saltwater aquaculture effluents.

Modern recirculating aquaculture systems (RAS) allowed culturing saltwater species everywhere, including locations away from marine water sources. However, one of the biggest concerns when operating these systems are the management and costs associated to the reduction/removal of nutrient loads present in effluent water, as saline nutrient rich effluent cannot be directly discarded/used in land to avoid salinization^8,9^. Unused nutrients that are commonly wasted in aquaculture effluents are composed by particulate organic matter (POM), dissolved organic matter (DOM) [including dissolved organic nitrogen (DON) and phosphorus (DOP)] and as dissolved inorganic nutrients (including dissolved inorganic nitrogen (DIN) = NO_x_-N + NH_4_-N and dissolved inorganic phosphorus (DIP) = PO_4_-P]^10,11^. The recovery of dissolved inorganic nutrients can be successfully achieved using primary producers as extractive species, such as microalgae, seaweeds and halophytes, while the recovery of POM can be pursued using deposit-feeders and filter-feeders, like polychaetes and bivalves^3,12,13^. The integration of multiple species from different trophic levels in the same IMTA design allows to recover particulate and dissolved nutrients in the form of valuable biomass of these extra crops. This has been previously described in studies addressing the combined use of bivalves and seaweeds^14–23^, bivalves and microalgae^24^, bivalves and halophytes^25^, echinoderms and seaweeds^26^, polychaetes and seaweeds^27^ and polychaetes and halophytes^11^. More than 50% of these studies were performed in the time-period between 2015 and 2020 evidencing that this is currently a hot-topic in aquaculture. In general, most IMTA designs addressed to date advocate the culture of different extractive species in separate tanks^11,15,17,24,25,28^, following the trophic relations, which consequently require a larger operational area. This is often pointed as one of the major constrains to successfully develop an IMTA framework for new or ongoing operations. Indeed, producers need to allocate part of the area defined for the aquaculture of the target species being farmed (e.g., finfish or crustaceans) to produce the extractive species, which often have a lower commercial value^29^. As an example, one can refer that in order to recover 10% of dissolved inorganic nitrogen produced by 1000–1800 ton of salmon in 1 ha, nearly 10–23 ha of seaweed culture area are needed^29–32^. Concerning halophytes, it was estimated that approximately 10.000 m^2^ of constructed wetlands planted with *Salicornia persica* would be required to recover N and total suspended solids (TSS) originated from 900 kg of 45% crude protein fish feed during one year (11 m^2^ Kg^−1^ of feed)^33^. In soilless production systems (e.g. aquaponics—using the deep water culture technique) it was estimated that a planted area of ≈ 14.4 m^2^ with *Salicornia dolichostachya* (1,128 plants–78 plants m^−2^) would be required to remove 189 g N and 29 g of P excreted by *Dicentrarchus labrax*^34^. In this way, giving that farmers commonly have limited farming areas to culture their target fed species, it is imperative to work on the development of IMTA designs where different extractive species can be produced using innovative approaches that minimize the required operational area.

Another remaining challenge, worth highlighting, is that several modern RAS use saline groundwater which is usually supersaturated with nitrogen, argon and carbon dioxide^35^. These compounds are harmful to the main species of finfish and crustaceans being produced, and in order to increase oxygen in the water to concentrations within optimum values^36^ degassing and aeration treatments must be used to pre-treat the water, safeguarding against harmful gases. Saline groundwater can also be supersaturated with iron, then treatments employing chemical oxidation are employed to promote iron oxides to precipitate and subsequently removed using rapid sand filtration or settling basins^36^. These pre-treatments, as well as others like ozonation, needed for the production of the fed species, can remove essential micro-nutrients that might negatively affect the production of extractive primary producers like halophytes.

Polychaete worms, such as *Hediste diversicolor* O.F. Müller, 1776 and *Arenicola marina* Linnaeus, 1758, can be key extractive species to recover wasted nutrients from POM present in aquaculture effluents, while halophytes such as *Salicornia ramosissima* J. Woods, 1851 can be key extractive species to recover dissolved inorganic nutrients (mainly nitrogen and phosphorus). Therefore, the present study evaluated the bioremediation performance and biomass production of the combined culture of polychaetes and halophytes, namely, *A. marina* and *S. ramosissima* (Amar + Sram) and *H. diversicolor* and *S. ramosissima* (Hdiv + Sram) using the effluent water from a shrimp RAS operated with pre-treated saline groundwater (*ca.* 20 g L^−1^ of salt). These different IMTA designs were tested using different operational areas designated as single polyculture tank (1 T) and as two trophic levels separated tanks (2 T) (0.3 and 0.6 m^2^ of operational area, respectively).

## Results

### Characterization of abiotic conditions and inflowing water composition

The average values of inflowing water abiotic conditions and composition monitored during the experiment are summarized in Table 1 (characterization over time—Supplementary Figs. S1a–d and S2a–c, respectively). Particulate organic matter (POM) and dissolved inorganic nitrogen and phosphorus (DIN and DIP) monitored in the inflowing water accounted for 75% of total suspended particulate matter (SPM), 70–75% of total nitrogen (TN) and 75–85% of total phosphorus (TP), respectively. During the polyculture trial combining polychaetes and halophytes (60 days) it was estimated that each tank filtered 490 L of effluent, which contained ≈ 5.6 g POM, 5.5 g TN (74% DIN) and 1.1 g TP (84% DIP). In the second period (60–120 days) where only polychaetes were maintained it was estimated that each tank filtered 462 L of effluent water, which contained ≈ 5.4 g POM, 6.9 g TN (74% DIN) and 1.4 g TP (75% DIP) (Supplementary Table S1).

**Table 1:** Abiotic conditions (pH, oxygen, temperature and salinity) and composition [suspended particulate matter (SPM), particulate organic matter (POM; %LOI in SPM), total nitrogen (TN), total phosphorus (TP), dissolved inorganic nitrogen (DIN) and phosphorus (DIP)] measured in inflowing water.

Parameter	Study period	
	1–60 days	61–120 days
	*Polychaetes* + *Halophytes*	*Polychaetes*
**Inflowing water abiotic conditions**		
pH	8.28 ± 0.07	8.25 ± 0.07
DO (mg L^-1^)	7.70 ± 0.48	8.52 ± 0.44
Temperature (°C)	23.81 ± 1.88	19.45 ± 2.44
Salinity (ppt)	18.90 ± 0.82	17.38 ± 2.57
**Inflowing water composition**		
SPM (mg L^−1^)	15.14 ± 4.04	15.89 ± 0.85
POM (mg L^−1^)	11.49 ± 2.85	11.70 ± 0.42
TN (mg L^−1^)	11.13 ± 3.91	14.86 ± 0.83
DIN (mg L^−1^)	8.25 ± 3.92	10.93 ± 0.35
TP (mg L^−1^)	2.24 ± 0.86	3.09 ± 0.66
DIP (mg L^−1^)	1.89 ± 0.84	2.32 ± 0.47

Average values ± SD (n = 5).

### Bioremediation of particulate organic matter (POM) and generation of polychaetes biomass

**Figure 1:**
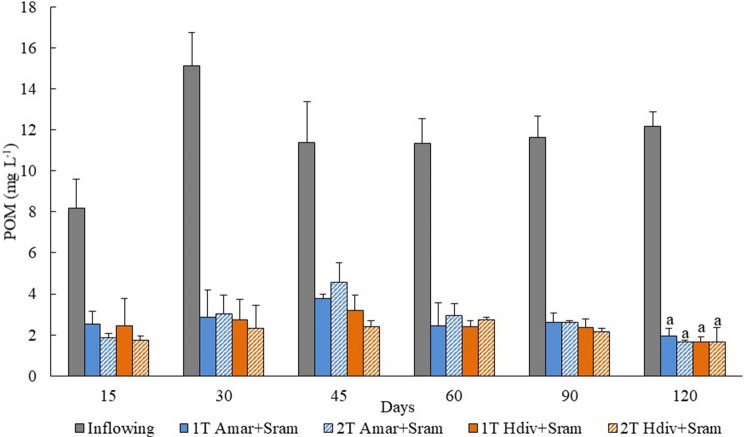
Particulate organic matter (POM) quantified in the inflowing and outflowing efluent of IMTA designs tested in the present study using as extractive species polychaetes (*Arenicola marina*—Amar—and *Hediste diversicolor*—Hdiv) and halophytes (*Salicornia ramosissima*—Sram) cultured in the same tank (1 T) or in two separate tanks (2 T). Average values ± SD (n = 5). Statistical analysis performed only at 120 days when the biomass of extractive species was evaluated. No significant differences (*p* < 0.05) between IMTA designs were observed.

The concentrations of POM quantified in the inflowing and outflowing water of 1 T and 2 T IMTA designs stocked with *A. marina* and *H. diversicolor* over the study period (120 days) are presented in Fig. 1. No significant differences were found in POM concentration monitored in outflowing effluent of different IMTA designs (*Two-way ANOVA*, *p* > 0.05), with the values corresponding to retention efficiencies between 84 and 87% of inflowing POM.

**Figure 2:**
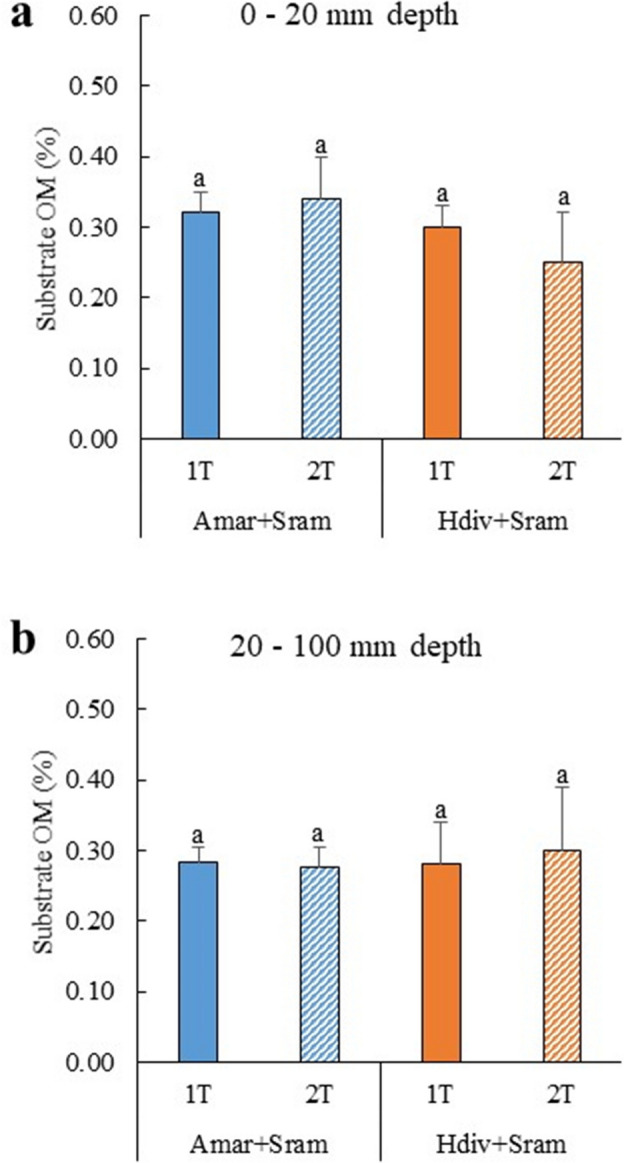
Organic matter (OM) content determined in the top 20 mm and 20–100 mm substratum layers of IMTA designs tested in the present study using as extractive species polychaetes (*Arenicola marina*—Amar—and *Hediste diversicolor*—Hdiv) and halophytes (*Salicornia ramosissima*—Sram) cultured in the same tank (1 T) or in two separate tanks (2 T). Average values ± SD (n = 5). No significant differences (*p* < 0.05) between IMTA designs were observed.

The concentration of OM (determined through loss of ignition—%LOI) quantified at the end of experiment in the top 20 mm of the substratum of polychaetes assisted sand filters (PASFs) stocked with *A. marina* and *H. diversicolor* are displayed in Fig. 2a,b. No significant differences between treatments were found in OM content monitored in the top 20 mm (*Post-hoc* Tukey HSD, *p* > 0.05) and 20–100 mm substratum layers (*Two-way ANOVA*, *p* > 0.05), with values ranging between 0.25–0.34 and 0.27–0.30% LOI, respectively.

**Figure 3:**
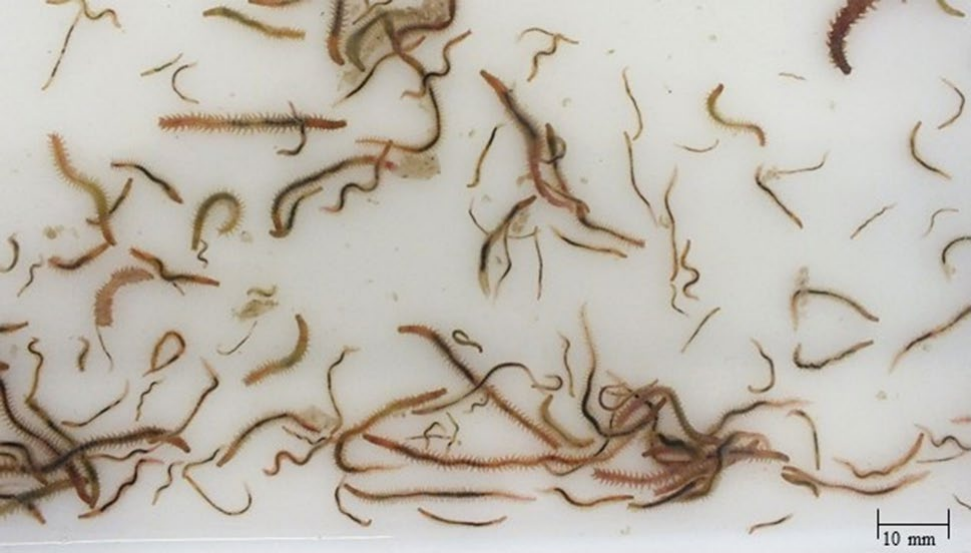
*Hediste diversicolor* juveniles produced after 120 days of culture.

The average values (± SD) of biomass and density of *A. marina* and *H. diversicolor* determined at the end of the experiment are displayed in Table 2. For *A. marina* mortalities between 90 and 95% were observed revealing that the experimental conditions impaired the successful culture of this polychaete species. Regarding *H. diversicolor,* the final densities obtained for both operational IMTA designs (1 T and 2 T) were ≈ 14–15 times that of initial values. These polychaetes corresponded to newly generated biomass of different sizes, including worms with 20–30 mm length (Fig. 3). The biomass of *H. diversicolor* was significantly higher to the one produced by *A. marina* independently of operational design tested (*Post-hoc* Tukey HSD, *p* < 0.05). Between 1 and 2 T designs stocked with the same polychaete species no significant differences were found in biomass produced (*Post-hoc* Tukey HSD, *p* > 0.05).

**Table 2:** Density and total biomass of polychaetes (*Arenicola marina*—Amar—and *Hediste diversicolor*—Hdiv) cultured in the same tank with halophytes (*Salicornia ramosissima*—Sram) (1 T) or in two separate tanks (2 T) at day 120. Average values ± SD (n = 5).

IMTA design	Density	Total biomass
	(ind. m^−2^)	(g m^−2^)
1 T Amar + Sram	3 ± 2	2.4 ± 2.7^a^
		(0.2 ± 0.3)
2 T Amar + Sram	5 ± 3	5.5 ± 3.9^a^
		(0.5 ± 0.4)
1 T Hdiv + Sram	3993 ± 1496	98.4 ± 25.7^b^
		(13.5 ± 3.7)
2 T Hdiv + Sram	4425 ± 540	77.5 ± 11.2^b^
		(10.8 ± 1.5)

Different letters indicate significant differences (*p* < 0.05) between IMTA designs. The values between brackets indicate the dry weight biomass.

### Extraction of dissolved inorganic nitrogen and phosphorus (DIN and DIP, respectively) and generation of *Salicornia ramosissima* biomass

**Figure 4:**
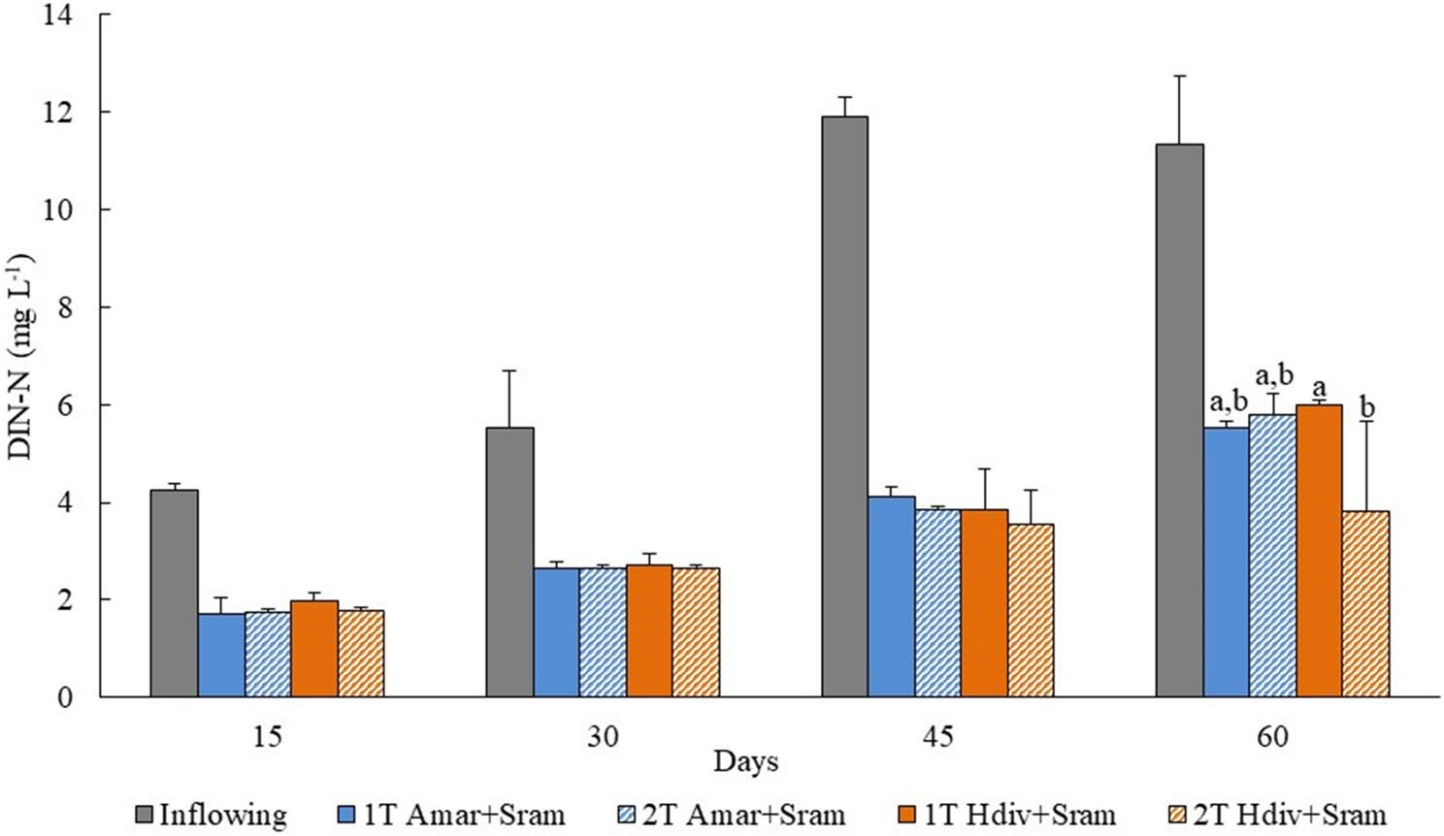
Dissolved inorganic nitrogen (DIN) quantified in the inflowing and outflowing effluent of IMTA designs tested in the present study using as extractive species polychaetes (*Arenicola marina*—Amar—and *Hediste diversicolor*—Hdiv) and halophytes (*Salicornia ramosissima*—Sram) cultured in the same tank (1 T) or in two separate tanks (2 T). Average values ± SD (n = 5). Statistical analysis performed only for the period of 120 days when biomass was evaluated. Different letters indicate significant differences (*p* < 0.05) between IMTA designs.

**Figure 5:**
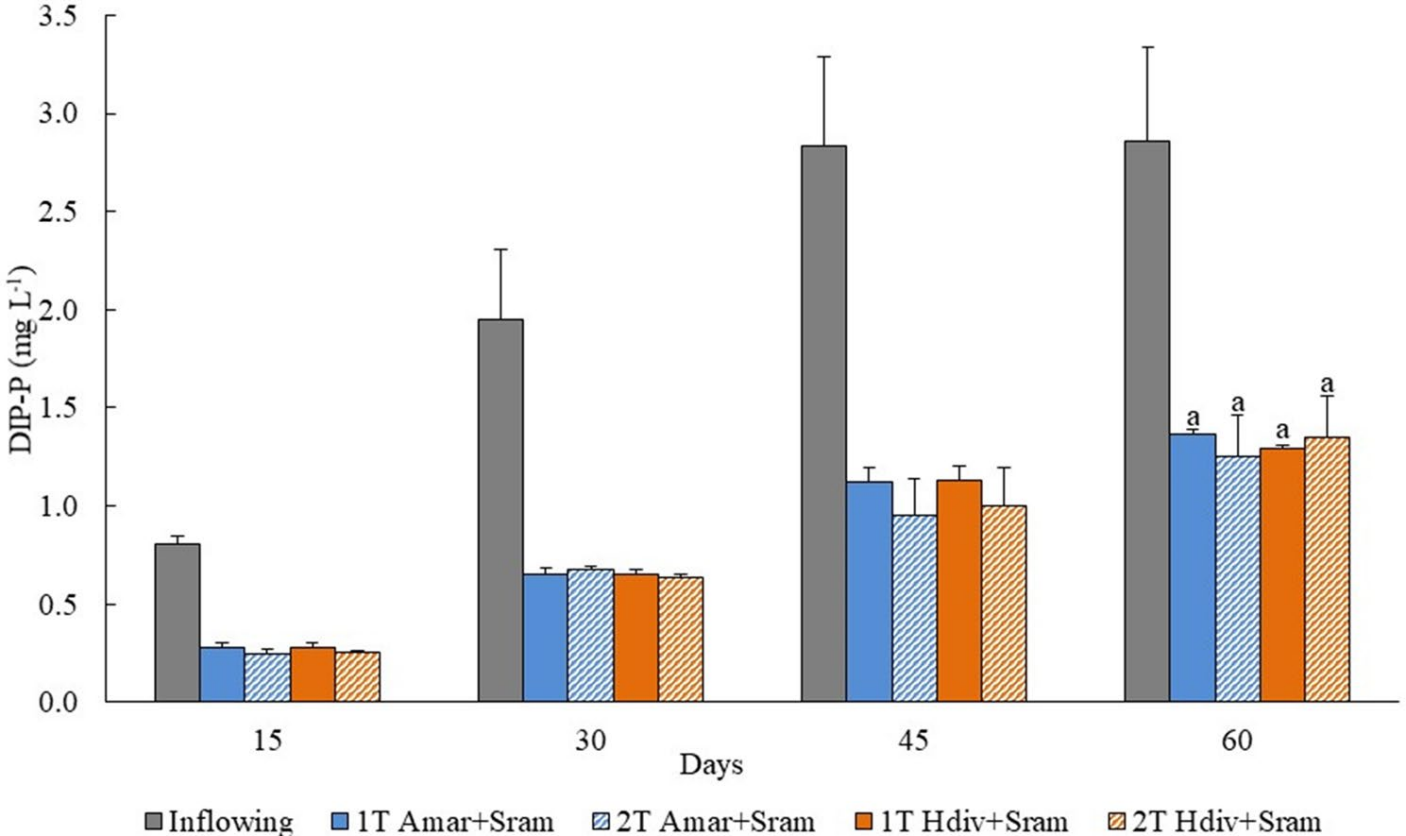
Dissolved inorganic phosphorus (DIP) quantified in the inflowing and outflowing effluent of IMTA designs tested using as extractive species polychaetes (*Arenicola marina*—Amar—and *Hediste diversicolor*—Hdiv) and halophytes (*Salicornia ramosissima*—Sram) cultured in the same tank (1 T) or in two separate tanks (2 T). Average values ± SD (n = 5). Statistical analysis performed only at 120 days when the biomass of extractive species was evaluated. No significant differences (*p* < 0.05) between IMTA designs were observed.

The concentrations of DIN and DIP monitored in the inflowing and outflowing effluent of 1 T and 2 T IMTA designs stocked with Amar + Sram and Hdiv + Sram at day 60 are displayed in Figs. 4 and 5, respectively. The DIN concentration monitored in outflowing water of 2 T Hdiv + Sram was significantly lower than the one exhibited by 1 T Hdiv + Sram (*Post-hoc* Tukey HSD, *p* < 0.05), while between remaining IMTA designs no significant differences were found (*Post-hoc* Tukey HSD, *p* > 0.05). Concerning DIP, no significant differences were found in concentrations measured in outflowing water of different IMTA designs (*Two-way ANOVA*, *p* > 0.05). Bioremediation efficiencies of 48–66% and 52–56% were observed for inflowing DIN and DIP, respectively.

**Figure 6:**
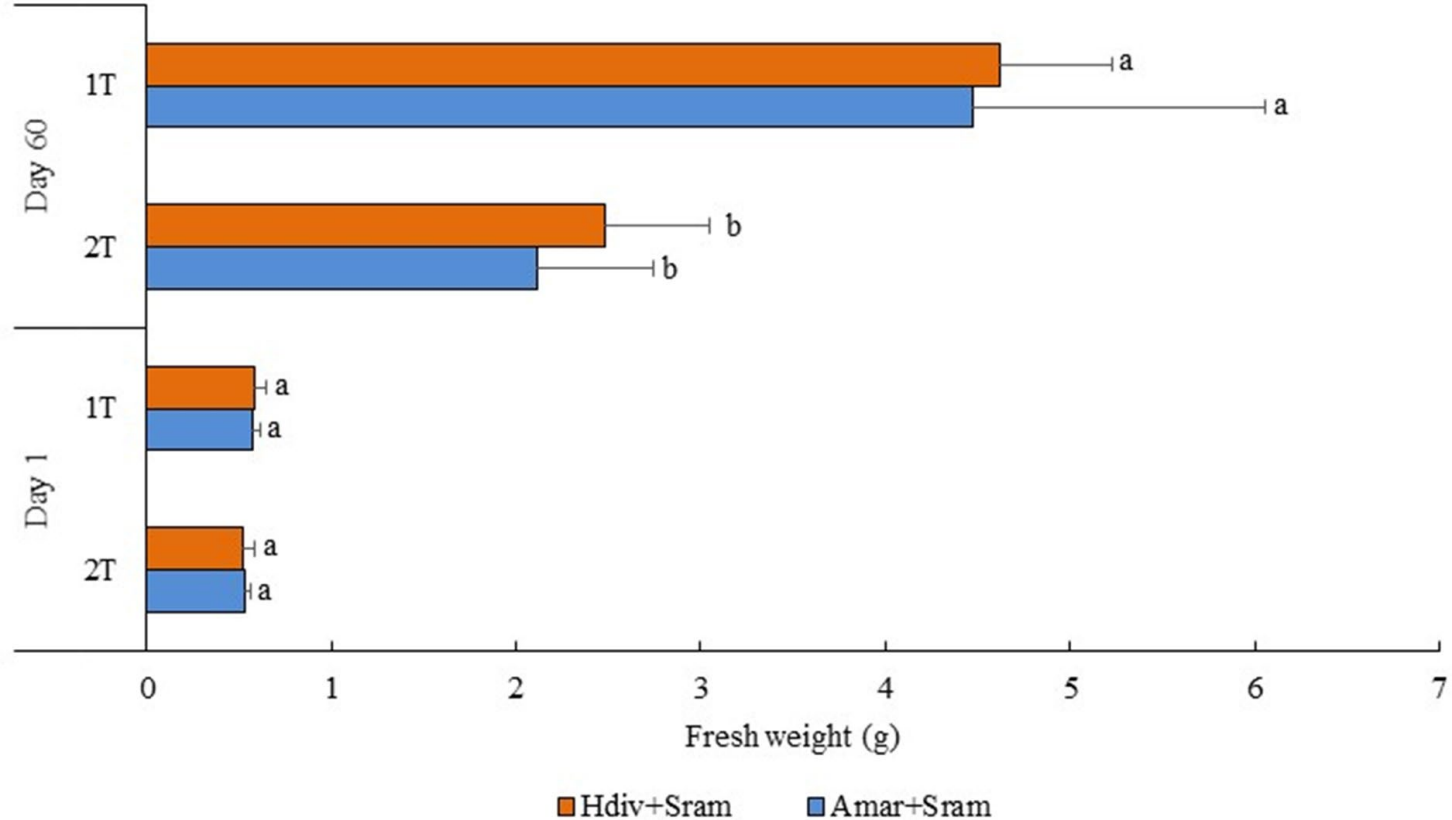
Fresh weight of *Salicornia ramosissima* (Sram) at day 1 and 60 cultured in the same tank (1 T) or in two separate tanks (2 T) than polychaetes (*Arenicola marina*—Amar—and *Hediste diversicolor*—Hdiv). Average ± SD (n = 5). Different letters in each time-period indicates significant differences (*p* < 0.05) between IMTA designs.

**Figure 7:**
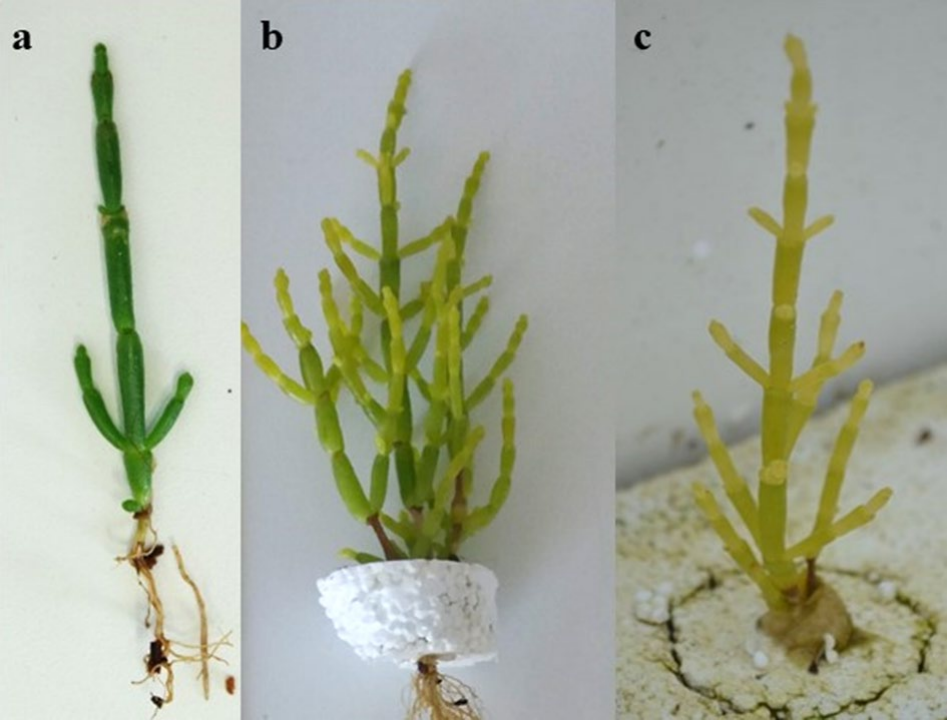
Evolution of coloration of *Salicornia ramosissima* over the experimental period: (**a**) plants at day 1; (**b**) plants at day 30 and (**c**) plants at day 60.

The average weight of *S. ramosissima* cultured under different IMTA designs stocked with Amar + Sram and Hdiv + Sram are displayed in Fig. 6. At day 60, the plants grown on 1 T designs revealed a significantly higher average weight (≈ 2-times higher) than the ones reported in 2 T designs independently of stocked polychaete species (*Post-hoc* Tukey HSD, *p* < 0.05). Table 3 summarizes the average values (± SD) of density and total biomass reported at day 60. In this period, 36–40% and 44–56% of the plants initially stocked in both 1 T and 2 T IMTA designs enter senescence and were considered not viable to biomass account, respectively. No significant differences were verified in final plant density reported in 1 T and 2 T designs (*Post-hoc* Tukey HSD, *p* < 0.05). The total plant biomass and inherent aboveground biomass generated by both 1 T designs were significantly higher than the ones reported in 2 T (*Post-hoc* Tukey HSD, *p* < 0.05), except between 2 T Hdiv + Sram and 1 T Amar + Sram (*Post-hoc* Tukey HSD, *p* > 0.05). The belowground biomass produced was higher in 1 T designs, with the values obtained in 1 T Amar + Sram being significantly higher than the ones obtained in 2 T Amar + Sram (*Post-hoc* Tukey HSD, *p* < 0.05). The final biomass reported in 1 T and 2 T designs was ≈ 4.0–5.1 and 1.9–2.5 times higher than the initially stocked values, respectively. The aboveground fresh weight (FW) biomass represented 70–80% of total plant biomass produced, while their dry weight (DW) biomass corresponded to approximately 8% of the FW value. The plants acquired a yellowish coloration over the study (Fig. 7a–c) with a large percentage of them going into senescence.

**Table 3:** Final density, total plant fresh weight biomass, aboveground and belowground fresh weight biomass of halophytes (*Salicornia ramosissima*—Sram) cultured in the same tank (1 T) or in separate tanks (2 T) with polychaetes (*Arenicola marina*—Amar—and *Hediste diversicolor*—Hdiv) at day 60. Average values ± SD (n = 5).

IMTA design	Density	Total plant	Aboveground	Belowground
	(Plants m^−2^)	(g m^−2^)	(g m^−2^)	(g m^−2^)
1 T Amar + Sram	49 ± 6^a^	194.2 ± 80.5^a,b^	148.6 ± 68.8^a,b^	53.2 ± 25.4^a^
		(15.8 ± 7.4)	(11.6 ± 5.4)	(4.3 ± 2.0)
2 T Amar + Sram	37 ± 9^a^	84.9 ± 34.8^c^	63.3 ± 26.3^c^	21.8 ± 8.8^b^
		(6.7 ± 2.7)	(4.9 ± 2.0)	(1.7 ± 0.7)
1 T Hdiv + Sram	54 ± 9^a^	225.6 ± 54.4^a^	171.1 ± 39.2^a^	54.6 ± 15.3^a^
		(17.7 ± 4.3)	(13.3 ± 3.1)	(4.4 ± 1.2)
2 T Hdiv + Sram	47 ± 14^a^	114.1 ± 30.1^b,c^	86.3 ± 22.2^b,c^	27.8 ± 7.8^a,b^
		(9.0 ± 2.3)	(6.7 ± 1.7)	(2.2 ± 0.6)

Different letters indicate significant differences (*p* < 0.05) between IMTA designs. The values between brackets indicate the dry weight biomass.

## Pigment profile of *Salicornia ramosissima* cultured under 1 T and 2 T IMTA designs

**Figure 8:**
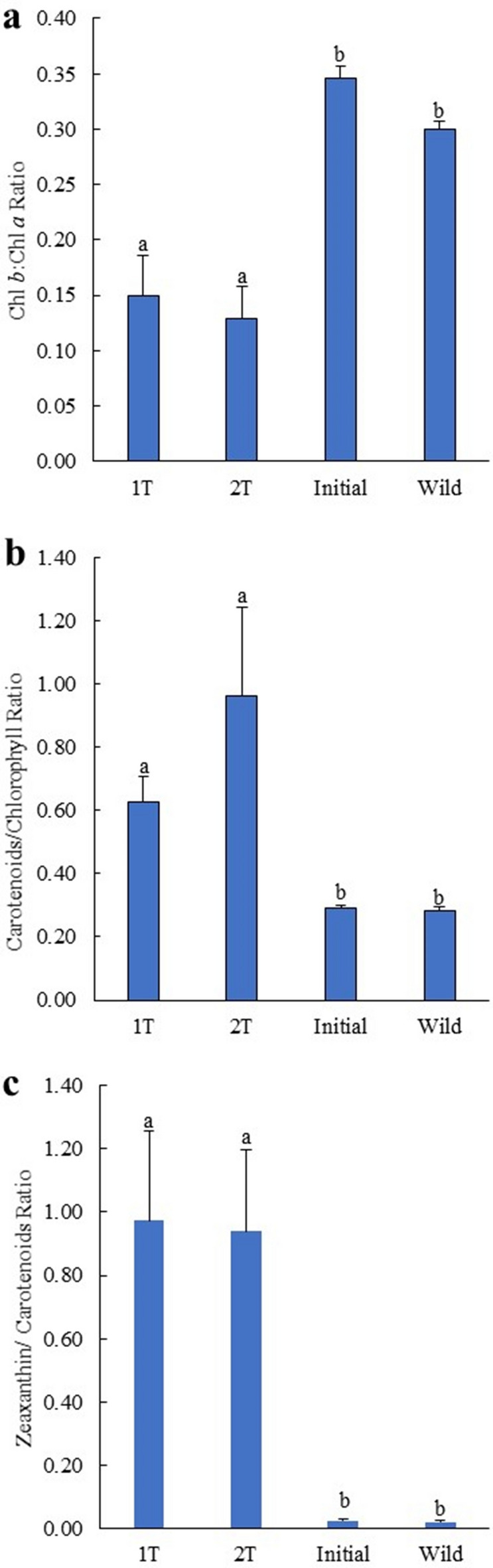
Chlorophyll *b*:chlorophyll *a* (Chl *b*/Chl *a*), total carotenoids/chlorophyll and zeaxanthin/carotenoids ratios measured in *Salicornia ramosissima* cultured in the same tank (1 T) or in two separate tanks (2 T) than polychaetes (*Arenicola marina* and *Hediste diversicolor*). Average values ± SD (n = 5). Different letters indicate significant differences (*p* < 0.05) between samples.

The pigments recorded in *S. ramosissima* initially stocked, cultured and collected from the wild were the carotenoids 9′-*cis*-neoxanthin, violaxanthin, antheraxanthin, lutein, zeaxanthin and β,β-carotene and the chlorophylls *a* and *b* (Chl *a* and Chl *b*) (Table 4). The average values (± SD) of pigment concentrations identified in *S. ramosissima* cultured under 1 T and 2 T IMTA designs, as well as the profile of initially stocked plants and wild conspecifics are displayed in Table 5. Concentrations of 9′-*cis*-neoxanthin, violaxanthin, lutein, β,β-carotene, Chls *a* and *b* were significantly higher in initially stocked and wild conspecifics compared to plants cultured under 1 T and 2 T (*Post-hoc* Tukey HSD, *p* < 0.05). On the other hand, significant higher concentrations of zeaxanthin were observed in plants cultured under 1 T and 2 T when compared to initially stocked and wild plants (*Post-hoc* Tukey HSD, *p* < 0.05). No significant differences in pigments profile was verified between plants cultured under 1 T and 2 T designs (*Post-hoc* Tukey HSD, *p* > 0.05). The Chl *b*/Chl *a* ratio of cultured plants was lower to the ones exhibited by initially stocked and wild conspecifics (≈ 2-times lower), while total carotenoids/chlorophyll and zeaxanthin/carotenoids ratios were higher (≈ 2.2–3.4 and 36–46-times, respectively) (Fig. 8a–c). Significant differences were found in the above-mentioned ratios between cultured and initially stocked and wild conspecifics plants (*Post-hoc* Tukey HSD, *p* < 0.05).

**Table 4:** List of pigments detected in halophytes (*Salicornia ramosissima*) with average retention times and absorption maxima (λ max).

	Retention time	λ max (nm)
9′-*cis*-Neoxanthin	12.96	416, 438, 467
Violaxanthin	14.17	417, 441, 472
Anteraxanthin	16.10	424, 448, 477
Lutein	17.74	425, 448, 476
Zeaxanthin	17.99	430, 454, 481
Chlorophyll *b*	22.83	458, 596, 646
Chlorophyll *a*	24.46	430, 617, 663
β,β-Carotene	28.78	430, 454, 480

**Table 5:** Pigment concentrations (µg g^−1^ DW biomass) recorded in halophytes (*Salicornia ramosissima*) cultured cultured in the same tank (1 T) or in separate tanks (2 T) with polychaetes (*Arenicola marina*—Amar—and *Hediste diversicolor*—Hdiv), as well as initially stocked plants and conspecifics from the wild. Average values ± SD (n = 5).

	1 T	2 T	Initial	Wild
9′-*cis*-Neoxanthin	2.8 ± 1.2^a^	2.3 ± 0.5^a^	73.7 ± 13.9^b^	58.8 ± 7.8^b^
Violaxanthin	7.4 ± 6.9^a^	5.0 ± 1.1^a^	182.7 ± 31.6^b^	183.8 ± 29.7^b^
Anteraxanthin	11.2 ± 3.6^a^	9.6 ± 1.9^a^	14.1 ± 2.9^a^	15.6 ± 3.5^a^
Lutein	31.0 ± 8.9^a^	29.1 ± 4.4^a^	353.7 ± 51.0^b^	259.6 ± 37.8^c^
Zeaxanthin	56.7 ± 14.7^a^	59.5 ± 7.8^a^	18.8 ± 4.1^b^	12.6 ± 2.3^b^
Chlorophyll *b*	25.5 ± 11.0^a^	16.1 ± 5.9^a^	661.4 ± 85.5^b^	508.2 ± 74.3^b^
Chlorophyll *a*	16.5.9 ± 33.5^a^	123.8 ± 34.3^a^	1912.1 ± 231.9^b^	1695.7 ± 262.8^b^
β,β-Carotene	9.8 ± 1.4^a^	9.0 ± 2.7^a^	106.8 ± 13.0^b^	95.1 ± 20.1^b^

Different letters indicate significant differences (*p* < 0.05) between samples.

## Discussion

This work aimed to compare the bioremediation performance and biomass production through the combined culture of *A. marina* and *S. ramosissima* (Amar + Sram) and *H. diversicolor* and *S. ramosissima* (Hdiv + Sram) using a single polyculture tank (1 T) and two trophic levels separated tanks (2 T), IMTA designs with different operational areas (0.3 and 0.6 m^2^, respectively). The experiment was performed outdoors, and great variability was verified for salinity and water temperature (*ca.* 12–20 g of salt per litre, 16–28 °C, respectively), while for pH and dissolved oxygen (DO) more stable values were reported (8.1–8.4 and 6.8–9.5 mg L^−1^, respectively). Despite the variability, all these parameters are within the range of values monitored in water and intertidal water pools of Aveiro coastal lagoon^37,38^, place of collection of polychaetes used in the present study.

Bioremediation efficiencies of 74–87% POM, 56–64% DIN and 60–65% DIP of inflowing effluent, were reported in 1 T and 2 T IMTA designs stocked with Amar + Sram and Hdiv + Sram. The OM in the top 20 and 20–100 mm substratum depth was reported between 0.2–0.4% LOI and no differences were verified in IMTA designs stocked with *A. marina* and *H. diversicolor*. Polychaete assisted sand filters are highly efficient in the retention of POM which is incorporated into valuable extractive worm biomass^11,39–42^. A very important role was played by the sand bed of PASFs by retaining and keeping OM available to sustain the growth of polychaetes, while these organisms promoted bioturbation (sediment re-working and bio-irrigation, which enables dissolved oxygen to reach deeper layers of the substratum), a feature which is paramount to avoid the clogging of the system and maintain the percolation of water through the sand bed^39^. This effect was clearly showed in the current study, as at the end of experimental period each of the designs tested stocked with polychaetes remained operational, while control tanks were clogged and water overflowed. The conversion of POM into valuable worm biomass is expected to occur in PASFs, although this was not the case for those stocked with *A. marina* which revealed mortalities above 90%. The biomass of this polychaete species was significantly lower than the one produced in designs stocked with *H. diversicolor*, however it is important to keep in mind that these differences result from the high mortality reported in 1 T and 2 T designs stocked with *A. marina*. When comparing the biomass of this polychaete (iteroparous species which reaches sexual maturity at 2–3 years of age) to the one produced by *H. diversicolor* which revealed a completely different life cycle (semelparous species which reaches sexual maturity at 1–2 years of age), we also have to keep in mind that in a 120-day study, at best, we would only be able to compare the biomass gain of initially stocked individuals of *A. marina* with the biomass gain resulting from juveniles of *H. diversicolor* produced in the same period. Although temperatures above 20 °C are frequently monitored in intertidal water pools^37,38^ next to wild stocks of *A. marina*, previous results showed that temperature above this threshold may have impaired the success culture of this polychaete species. For example, in a previous study carried out with temperatures between 12 and 20 °C which aimed to evaluate the viability of *A. marina* under IMTA conditions, the survival reported was higher than 90% at a density of ≈ 150 ind. m^−2^; these worms were fed with fish waste and displayed a considerable increment of biomass (from 80 to 480 g m^−2^ during 55 days)^43^. In another study, the best growth performances of these worms were achieved when they were provided with a formulated fish feed and salmon faeces (growth rate ≈ 32% and 23% after 14 and 39 days, respectively), outgrowing conspecifics fed with other diets (e.g. fresh seaweed and organic matter in sediment without additional feed)^44^. The culture of this polychaete species was also evaluated using a substrate containing 25% of mud from aquaculture and 75% sand, with worms revealing an average growth of 106% after 39 days^45^. Mortalities reported in the two last studies referred were likely linked with water temperature also being recorded above optimal values, thus reinforcing the need to strictly control this parameter when aiming to culture *A. marina* outdoors.

Concerning *H. diversicolor*, the culture conditions employed proved to be adequate for their development, with the final densities recorded in 1 T and 2 T IMTA designs being approximately 15 times that of the initial stocking densities (final density ≈ 4000–5000 ind. m^−2^). No significant differences were found between *H. diversicolor* FW biomass obtained in 1 T and 2 T designs, with the values obtained at the end being lower than the one present at the beginning of experiment (≈ 77–100 vs 130 g m^−2^, respectively). Nonetheless, it is important to highlight that the specimens at the end of the experiment corresponded to a newly generated population of juveniles that was just starting to grow and yet to achieve commercial size. *Hediste diversicolor* is characterized by a single reproductive episode before its death (being a semelparous species) and in the literature it is possible to find studies performed during longer periods which evaluated the productivity in terms of juvenile biomass originated from initially stocked polychaetes. For example, in a study performed over a longer period (≈ 150 days), similar results to the ones recorded in the present study were obtained in terms of increment of polychaetes density, from ≈ 400 to 7000 ind. m^−2^, in PASFs stocked with *H. diversicolor* being supplied an organic-rich effluent generated by a super-intensive commercial RAS producing *Solea senegalensis*^11^*.* In this last-mentioned study, a combined culture of polychaetes and halophyte plants (*Halimione portulacoides*) was performed, with extractive species being cultured in separate tanks and contributing to remove 70% of POM and 65% of DIN, respectively. As mentioned before, the culture in separate tanks require a larger operational area, which is often pointed as one of the major constraints to successfully develop IMTA framework for new or ongoing operations^29^*.* On studies performed over shorter periods (less than 60 days), this polychaete species was tested under different culture densities (250–2000 ind. m^−2^; 5–40 g m^−2^) to bioremediate the solid waste generated from tanks stocked with rainbow trout (*Oncorhynchus mykiss*), with increments of polychaete biomass between 2.4 and 6-times the initial values reported (29.7–96.07 g m^−2^)^42^. This polychaete species was also tested during 8 weeks on the bioremediation of effluents generated by the farming of great sturgeon (*Huso huso*), with a decrease of density from 2.000 ind. m^−2^ to ≈ 1.510 ind. m^−2^ and an increase of biomass gain of ≈ 233 g m^−2^ (SGR ≈ 3.4% d^−1^) being reported^46^.

Concerning the growth performance of halophytes, a significant higher average weight (≈ 2-times higher) was reported for plants cultured under 1 T design, independently of the polychaete species being stocked. The total biomass reported in 1 T and 2 T designs after 60 days of experimental trial accounted for 5–5.7 and 1.7–2.4-times higher than the initially stocked biomass, representing productivities of edible aboveground biomass of ≈ 150–170 and 60–90 g m^−2^, respectively. These values were lower than the ones obtained in previous works, such as for *S. bigelovii* cultured under hydroponic conditions at a density 3-times higher than the ones used in the present study (seedlings with ≈ 30 mm height planted at a density of ≈ 260 plants m^−2^) featured a marketable yield ≈ 1.7 kg m^−2^ after 28 days^47^. In another study, a productivity of ≈ 13 kg (≈ 0.9 kg m^−2^) was reported for *S. dolichostachya* (at ≈ 38 plants m^−2^) cultured in a zero-water-exchange RAS-IMTA (culture area—4.8 m^2^) during a 35-days trial^34^. In the present study, the culture conditions impaired *S. ramosissima* development. Very early on the trial, plants started to develop a yellowish coloration, with some even exhibiting evident signs of senescence. At the end of experimental period, approximately 40 to 60% of the plants were no longer viable, with this percentage being slightly higher in the 2 T IMTA design. The development of the yellowish coloration was most likely related to a lack of iron, as all the saltwater used in the shrimp farming system and RAS-IMTA design was pumped from a borehole and pre-treated through chemical oxidation. This treatment promotes the precipitation and removal of iron, along with other elements such as Mg, P, Ca and Mn^48,49^. These precipitates are then rapidly removed through the action of sand filters^36^. The development of a yellowish coloration was previously reported for *Tripolium pannonicum* cultured under aquaponic conditions in a zero-water-exchange RAS-IMTA^34^ and for *S. dolichostachya* cultured at very low salinities (0–5 mM NaCl) under hydroponic conditions^50^. These results may reveal a lack of key elements (micronutrients) to promote plant growth, such as Fe, Zn and Ca, which may be biofortified through fertilizers^51^. Another explanation for the yellowish coloration and premature senescence may be associated with the fact of the nutrient rich water employed in this study being stored for 3 days in a reservoir tank without aeration (a submerged pump only mixed the water 5 min every hour). These conditions may have favoured the production of toxic gases, such as hydrogen sulfide (H_2_S), which in plants has already proven to be a crucial player in the regulation of plant growth, development, and senescence^52^. However, it is important to note that plants of the genus *Salicornia* occur in wild predominantly at lower marshes where anoxic conditions were found^53^ and the sulfide accumulation can be high^54^. Plants cultured in the 1 T and 2 T IMTA designs exhibited lower content of chlorophylls *a, b* and total carotenoids (≈ 124–166, 16–26 and 118–128 µg g^−1^ DW biomass, respectively) than the ones recorded when they were initially stocked, as well as in conspecifics from the wild (≈ 1912–1695, 508–661 and 625–749 µg g^−1^ DW biomass, respectively). This decrease in pigment content may also have been caused by the use of borehole water pre-treated with chemical oxidation. In previous works it was found that the halophyte *T. pannonicum* displayed a significantly lower content of total chlorophyll and carotenoids when exposed to a media without iron supplementation (≈ 49 and 21 µg g^−1^ FW biomass, respectively) than conspecifics supplemented with this element (≈ 388–875 and 79–159 µg g^−1^ FW biomass, respectively)^49^. The higher levels of zeaxanthin quantified in cultured plants are probably the result of the activation of the violaxanthin cycle, a two-step cycle in which violaxanthin is converted first to antheraxanthin and the latter pigment is converted to zeaxanthin. The activation of this cycle is photoprotective and activated by high light intensity, but it can also be triggered by other abiotic stressors (e.g. anoxia and high temperature)^55^. On the other hand, these high levels of zeaxanthin are worth further investigation, as this carotenoid plays a critical role in the prevention of age-related eye diseases^56^. Halophytes displaying enhanced levels of zeaxanthin may likely fetch higher values in the functional foods market.

In general, the experimental design 1 T exhibited the best performance (i.e., similar bioremediation and polychaetes productivities and the best halophyte productivity). Moreover, it also allows to reduce by half the operational area required to implement an IMTA framework using these extractive species. The present study also revealed the significant limitations inherent to the culture of certain extractive species outdoors, namely when key abiotic conditions, such as water temperature, are difficult to control. In the present study, failing to control this parameter may have impaired the successful culture of *A. marina*. On the other hand, our study also showed that effluents from culture systems using brackish groundwater that has been treated to remove iron through chemical oxidation and rapid sand filtration may impair the use of some extractive species for IMTA. Indeed, the lack of iron (and eventually also other trace elements removed during chemical oxidation and rapid sand filtration) may be a bottleneck impairing the successful production of *S. ramosissima* and other valuable halophyte plants*.*

## Materials and methods

### Selected extractive species

All extractive species tested in the present work can be easily collected and are highly abundant in the study site, Ria de Aveiro coastal lagoon (Portugal 40°44′21.1"N 8°39′ 40.1″ W). A brief description of the main features of each extractive species is presented below:

#### Polychaete worms

The polychaete *H.* diversicolor, commonly known as ragworm, was selected to the present study due to its wide distribution along the shallow marine and brackish waters of European and North American estuaries and by being an infaunal species that creates a three-dimensional burrow network in sandy-mud bottoms^57^. Its “bentho-pelagic” life cycle is characterized by females brooding their embryos in the maternal burrow, the same location where its short pelagic larval life also takes place^58^. In the reproduction of this species, there is the rupture of the dissepiment in the female and by nephridies or rupture of the dissepiment in the male for gamete release; reproduction of this species is therefore followed by death of parental worms (semelparous species)^58^. The maturity takes between 1 and 2 years before spawning^58^. It is considered an active predator that displays omnivorous feeding habits, being ranked within the deposit-feeders polychaetes functional group^59^. Its bioturbation potential and high-valued biomass (rich in essential fatty acids)^42,46,59,60^ makes this polychaete a well-suited species for IMTA.

The polychaete *A. marina*, commonly known as lugworm, was selected to the present study due to its wide distribution in north-western European coasts, from the British Isles to the Iberian Peninsula, with its southern limit of distribution being close to 40° N^61^. It is found from middle to lower shores and reaches high abundances in sheltered estuarine sediments where it lives in U or J-shaped burrow (0.2–0.4 m deep)^61^. This species reproduces several times throughout its life cycle (iteroparous species) attaining sexual maturity at 2–3 years of age, having its sexes separated and displaying external fertilization, with different populations releasing eggs and sperm in a synchronized period of 2 weeks that runs from October to November^62,63^. It feeds on debris and microorganisms present in the sediment it ingests, leaving a characteristic depression on the top of the sediment (the “blow hole”) and later, after absorbing all organic content, releasing a characteristic worm cast^64^. In the wild, these polychaetes can reach densities between 100–150 ind. m^−2^ and tolerate salinities from 12 to 35^64^. Adults can reach between 120 and 200 mm in length, with lugworms being considered a premium bait for sea anglers^63,64^. The bioturbation promoted by this species, along with a growing interest in the biotechnological use of its biomass (e.g., production of extracellular hemoglobin [HBL Hb] as a promising substitute for human blood^65^ and use in solutions for organ preservation^66^) makes this polychaete species a promising candidate for IMTA.

The integration of both the above-mentioned polychaete species aimed to evaluate the performance of PASFs (i.e., bioremediation and biomass generation) stocked with two species that exhibit contrasting life cycles, distinct bioturbation strategies and biochemical profile.

#### Halophyte plants

*Salicornia ramosissima* is an halophyte plant popularly known as green samphire. It is widely distributed in the salt marshes of the Iberian Peninsula, western France and Serbia. It is an annual species that exhibits the best growth performances at low salinities, although it is able to tolerate high salinities^49,67,68^. These plants are considered a gourmet product for human consumption with their fresh branch tips being highly appreciated fresh^68–71^. Due to its saline content, this plant is also used dehydrated and grounded in preparations were it replaces traditional salt as green salt^71,72^. The nutritional profile of *S. ramosissima* is suitable for human consumption, revealing high protein content (5.20 g/100 g DW), *n*-3 and *n*-6 polyunsaturated fatty acids (mainly α-linolenic and linoleic acid)^68,71^ and minerals (such as sodium, potassium, calcium, magnesium, iron and manganese)^68^. These plants also exhibit a significant antioxidant and anti-inflammatory potential due to their total phenolics content^68,73^. In addition, seeds of *Salicornia* spp. contain considerable levels of oil and protein (e.g. *Salicornia bigelovii* seeds present 26–33% oil and 31% protein^74^). Oil yielding crop plants are very important for economic growth of the agriculture sector, with many of the fatty acids identified in plant seeds being highly demanded for several industrial sectors (e.g. plastics, textile, pharmaceuticals, cosmetics)^75,76^. The bioremediation potential exhibited by *Salicornia* spp.^33,34,77,78^ associated with the potential to produce valuable extractive biomass makes these plants key candidates for IMTA.

### IMTA design

The present study was performed from June to September 2018 at RiaSearch Lda. (Portugal), a research company in the field of aquaculture nutrition operating in the coastal lagoon Ria de Aveiro (40° 44′ 21.1″ N 8° 39′ 40.1″ W). The company employs RAS to grow Pacific white shrimp (*Penaeus vannamei*) and operates with brackish groundwater that is pre-treated through chemical oxidation and rapid sand filtration to remove iron. Shrimp were fed twice a day with a commercial diet for flatfish that present 62% of crude protein, 18% of crude fat and 0.3% of crude fiber (WINFlat—SPAROS). Uneaten feed and faeces that were siphoned from culture tanks were collected and concentrated in a reservoir (130 L) equipped with a pump programmed to work 5 min per hour in order to promote homogenization and avoid anaerobic conditions. The water concentrated in this reservoir was added every 3 days to an outflowing tank (0.34 m^−3^), from which it was pumped through a plastic trickling tower installed above the inflowing tank (0.34 m^−3^). From this inflowing tank the water was directed to the tanks where the different IMTA designs using polychaete assisted sand filters (PASFs) and halophytes in aquaponics were performed.

**Figure 9:**
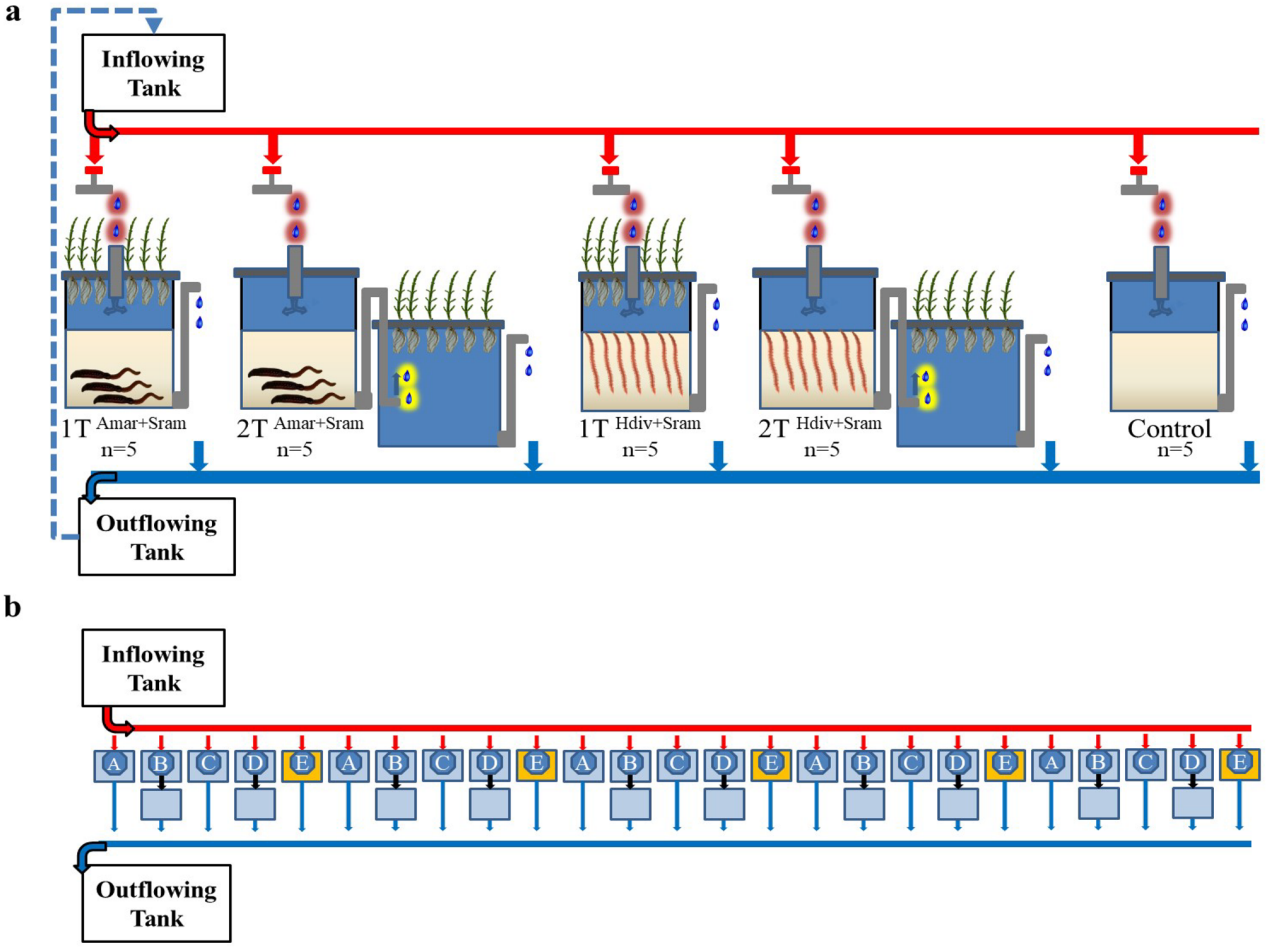
Schematic representation (1a) and distribution (1b) of different IMTA designs tested in the present study using as extractive species polychaetes (*Arenicola marina*—Amar—and *Hediste diversicolor*—Hdiv) and halophytes (*Salicornia ramosissima*—Sram) cultured in the same tank (1 T—designs A and C) or in two separate tanks (2 T—designs B and D): (A) 1 T Amar + Sram; (B) 2 T Amar + Sram; (C) 1 T Hdiv + Sram; (D) 2 T Hdiv + Sram; and (E) control tanks with no extractive species.

A schematic representation of the different IMTA designs tested in the present study is displayed in Fig. 9a. Designs 1TAmar + Sram and 2TAmar + Sram were stocked with the polychaete *A. marina* and the halophyte *S. ramosissima*, while designs 1 T Hdiv + Sram and 2 T Hdiv + Sram were stocked with the polychaete *H. diversicolor* and the halophyte *S. ramosissima*. Designs with 1 T cultured polychaetes and halophytes in the same tank (operational area ≈ 0.3 m^2^), with the roots of *S. ramosissima* being maintained in the water column of PASFs. Designs with 2 T cultured both extractive species in separate tanks (operational area ≈ 0.6 m^2^) with the water passing through the sand bed then being directed to the aquaponics unit. In 1 T designs, inflowing water entered PASFs tanks through a pipe whose bottom ended 0.1 m below the halophyte support plate to protect the roots from particulate organic matter. In 2 T designs, this configuration was also adopted to safeguard similar conditions. Each of the four IMTA designs performed in this study was evaluated using 5 replicates, with these being distributed as represented in Fig. 9b. Five control units with no polychaetes and no halophytes were also included in the experimental design. Each PASFs tank presented a volume of 0.05 m^3^ and a surface area of 0.3 m^2^ and was formed by a 150-mm sand column (0.5–0.7 mm grain size) in the bottom of the tank and a superficial 150-mm water column. These tanks were equipped with a bottom draining pipe to allow a complete percolation of water through the sand column. The aquaponic tank used in 2 T designs presented a water volume of 0.05 m^3^ and a surface area of 0.3 m^2^. Each tank of the different IMTA designs tested received a continuous water flow of 25 L h^−1^ (0.5 renewal each hour), with outflowing water being redirected to the general outflowing tank and recirculated. The system assembled to perform the present study displayed a total volume of 2 m^3^ and, every week, approximately 3% of this volume was added as fresh water to compensate for evaporation losses.

### IMTA extractive species culture and monitoring

Wild specimens of *A. marina* and *H. diversicolor* were collected in the coastal lagoon Ria de Aveiro by local fisherman. Culture tanks were inoculated with an initial stocking density of ≈ 67 ind. m^−2^ *A. marina* and 290 ind. m^−2^ of *H. diversicolor* (≈ 130 g FW m^−2^ for both polychaete species). Polychaetes were randomly distributed over the different IMTA designs 15 days before the beginning of the experiment for acclimation.

All plants of *S. ramosissima* used in the present work were germinated at RiaSearch Lda. Seeds were sown in trays containing a mix of coconut fibre and sand and were always kept outdoors under natural conditions of photoperiod and temperature. For 3 months, the coconut fibre was maintained wet through irrigation with ground brackish water pre-treated with chemical oxidation and rapid sand filtration seawater at a salinity 20 g L^−1^. After this period, plants with similar weight (0.5–0.6 g) were randomly selected and distributed over each tank of the different IMTA designs (25 plants per tank = 83 plants m^−2^) to start a two-week acclimation period.

Plants cultured in aquaponics were harvested 60 days after the beginning of the experiment to determine total plant biomass, as well as edible aboveground (shoots) and belowground biomass (roots). Due to the detection of *H. diversicolor* larvae in PASFs 60 days after the beginning of the experiment, the experimental period was prolonged for another 60 days (for a total of 120 days in total) but without any halophytes. During these additional 60 days, the addition of nutrient-rich water, as well as all monitoring and maintenance routines were performed exactly as during the first 60 days. At the end of experiment (120 days) the entire sand column of each PASFs was sieved and immediately transported to laboratory under refrigerated conditions where polychaetes from both species were sorted, counted and weighted.

### IMTA monitoring

During the whole experimental period, pH, temperature, dissolved oxygen (DO) and salinity were monitored weekly in the inflowing water of the experimental set up using a multiparameter probe (Lovibond SensoDirect 150). Samples from inflowing and outflowing water from each replicate of the four IMTA designs being tested were collected after 15, 30, 45, 60, 90 and 120 days of the beginning of the experiment. Samples of water entering all IMTA designs were collected after the nutrient rich water stored in the reservoir tank had been added to the outflowing tank and homogenized for at least 20 min prior being supplied to the inflowing tank (total volume of nutrient rich water = 0.68 m^3^). The samples of water exiting 1 T and 2 T designs were collected 2 and 4 h after the addition of nutrient-rich water, respectively, being these the times required to complete a full renewal cycle, respectively. The following parameters were determined at all sampling days: suspended particulate matter (SPM), particulate organic matter (POM), total nitrogen (TN) and phosphorus (TP) and dissolved inorganic nitrogen (DIN = NOx-N + NH_4_-N) and dissolved inorganic phosphorus (DIP = PO_4_-P). After the harvesting of the whole plant biomass (at day 60), DIN and DIP were no longer determined. All water samples were transported to the laboratory under dark and refrigerated conditions and immediately filtered (Whatman GF/C, Ø 47 mm dehydrated (105 °C) and pre-weighed filters) and subsequently frozen (− 20 °C) until further analysis. Water analysis were performed using an automated continuous flow analyser (Skalar San ^++^) to determine TN, TP, NH_4_-N, NOx-N and PO_4_-P. The analytical quality control was ensured by using calibration curves that were calculated from running standard solutions at the beginning and in parallel with blanks and samples. Filters containing SPM were processed following the EPA method 160.2. Samples from control tanks were not considered, as during the study period the sand in the bottom of these tanks was clogged with particulate material and water overflowed. For this reason, no comparison of bioremediation performance was possible. Sediment samples from each sand filter were collected in triplicate at the beginning and at the end of experiment to determine the organic matter (OM) content in the sediment. This determination was performed using the loss of ignition method (LOI%; 5 h combustion at 450 °C of substratum previously dried at 90 °C, until a constant weight was achieved).

### Determination of photosynthetic pigments of *Salicornia ramosissima*

Samples from the edible aerial part of *S. ramosissima* (n = 5) were collected from the four IMTA designs after 60 days, with samples from 1 T treatments being pooled (Amar + Sram and Hdiv + Sram), as well as those from 2 T treatments (Amar + Sram and Hdiv + Sram). Samples were also collected from plants used at the beginning of the experiment (n = 5) and from conspecifics collected from the wild (Ria de Aveiro coastal lagoon) (n = 5). The collection of halophyte plants from the wild was performed in compliance with current Portuguese and EU guidelines, legislation and codes of good practices framing the collection of living resources from the wild. All samples were frozen in liquid nitrogen and kept at − 80 °C until freeze-dried. Samples were grounded with mortar and pestle and 7–8 mg were weighed into Eppendorf tubes. Pigments were extracted using 0.5 mL of 95% cold buffered methanol (2% ammonium acetate), followed by 45 s sonication and 20 min incubation at − 20 °C in the dark. Extracts were filtered through 0.2 μm PTFE membrane filters and 50 µL were immediately injected into a HPLC system with a photodiode array detector SPD-M20A (Shimadzu, Kyoto, Japan). Chromatographic separation was carried out using a Supelcosil C18 column (25 cm length; 4.6 mm diameter; 5 µm particles; Sigma-Aldrich, St. Louis, MO, USA) following Mendes et al. (2007)^79^. Pigments were identified from absorbance spectra and retention times and concentrations were calculated using linear regression equations obtained from pure crystalline standards (DHI, Hørsolm, Denmark).

### Statistical analysis

To evaluate the existence of significant differences in the bioremediation performance (POM, DIN and DIP concentration in outflowing water and OM present in PASFs substratum) and the production of biomass (polychaetes: final biomass; halophytes: final biomass, density, average weight, aboveground and belowground biomass) from the four different IMTA designs (n = 5) two-way analysis of variance (ANOVA) were performed with polychaete species (two levels—*A. marina* and *H. diversicolor*) and operational area (1 T and 2 T) being used as predictive factors. To evaluate the existence of significant differences in the pigments concentration and pigments ratios exhibited between *S. ramosissima* cultured in 1 T and 2 T designs, in plants initially stocked in the experimental system and conspecifics collected from the wild (n = 5) a one-way analysis of variance (ANOVA) was performed with biomass source (4 levels—1 T, 2 T, initial and wild) being used as predictive factor. Data were previously checked for normality and homogeneity of variances through Anderson–Darling, Bartlett’s and Levene’s tests. *Post-hoc* Tukey’s HSD tests for individual means comparison were performed whenever significance was observed. When a condition of normality was not verified, the hypotheses were tested using Johnson transformed data. Significant differences were always considered at *p* < 0.05.

All the above-mentioned statistical analysis was performed using Minitab 18 Statistical Software (State College, PA). The statistical results of the tests mentioned above are summarized in supplementary Tables S2–S7.

## Supplementary Information

Supplementary Information 1.

Supplementary Information 2.

## Data Availability

All data generated or analysed during this study are included in this published article and its Supplementary Material files.
