# Supplementary material for: Recovering wasted nutrients from shrimp farming through the combined culture of polychaetes and halophytes
Source: Sci Rep. 2021 Mar 23;11:6587. doi: 10.1038/s41598-021-85922-y (PMC7988113; doi:10.1038/s41598-021-85922-y)
Supplement: Supplementary file 1 — Supplementary Information 1. [file 41598_2021_85922_MOESM1_ESM.docx]

**Supplementary Information for**

**Recovering wasted nutrients from shrimp farming through the combined culture of polychaetes and halophytes**

Daniel Jerónimo^1*^, Ana Isabel Lillebø^1^, Javier Cremades^2^, Paulo Cartaxana^1^, Ricardo Calado^1*^

*Corresponding authors:

E-mail: [danieljeronimo@ua.pt](mailto:danieljeronimo@ua.pt); Tel.: +351938547866 (D Jerónimo)

E-mail: [rjcalado@ua.pt](mailto:rjcalado@ua.pt); Tel.: +351234370779 (R Calado)

**
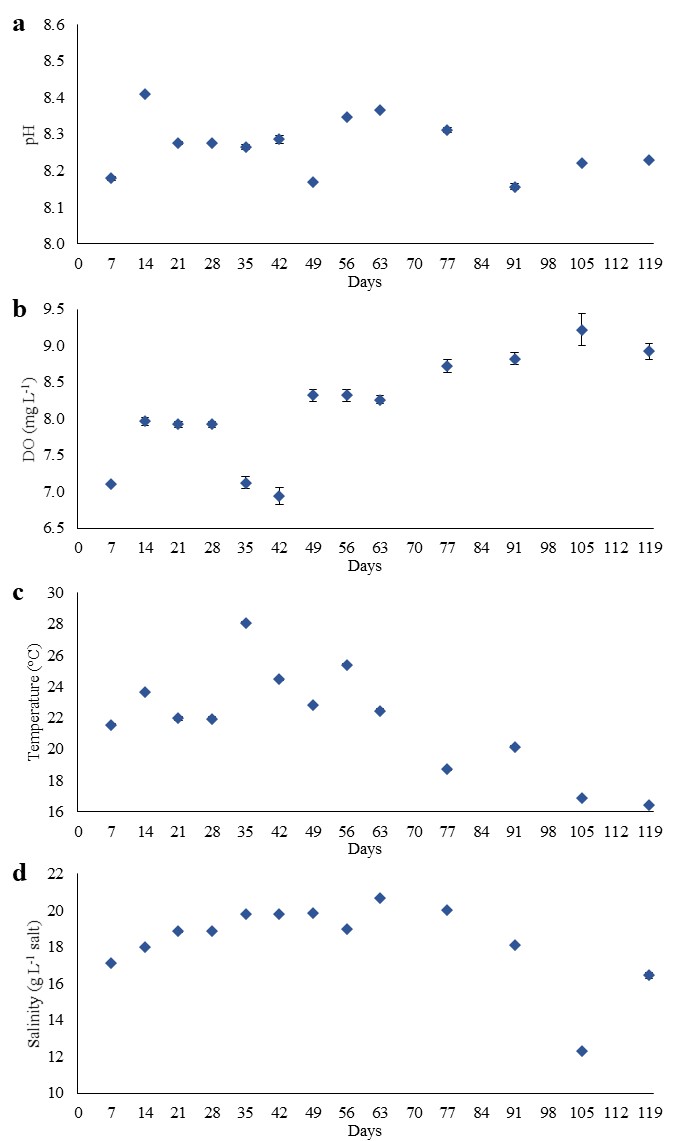
**

Figure S1. pH (S1a), dissolved oxygen (S1b), temperature (S1c) and salinity (S1d) measured in the inflowing water supplied to IMTA designs over the study period. Average values (±SD) (n=5).


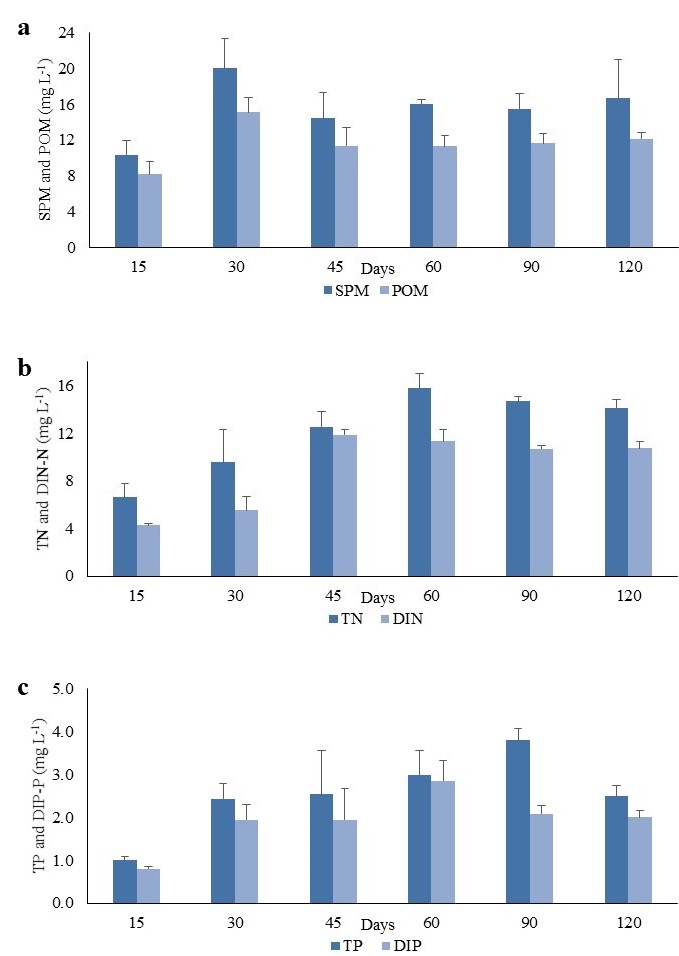


Figure S2. Suspended particulate matter (SPM) and particulate organic matter (POM) (S2a), total nitrogen (TN) and dissolved inorganic nitrogen (DIN-N) (S2b) and total phosphorus (TP) and dissolved inorganic phosphorus (DIP-P) (S2c) measured in the inflowing water supplied to IMTA designs over the study period. Average values (±SD) (n=5).

Table S1. Total water supplied to RAS-IMTA (sum of the whole outflowing and inflowing water volume of culture tanks) and estimation of the water volume entering each tank and associated particulate organic matter (POM), total nitrogen and phosphorus (TN and TP) and dissolved inorganic nitrogen and phosphorus (DIN-N and DIP-P). Estimate of water composition is supported by data displayed on Table 1.

| **Period (Days)** | **Total water supplied RAS-IMTA (L)** | **Total water supplied per tank (L)** | **POM (g)** | **TN (g)** | **DIN-N (g)** | **TP (g)** | **DIP-P (g)** |
| --- | --- | --- | --- | --- | --- | --- | --- |
|  |  |  |  |  |  |  |  |
| 0 - 60 | 12240 | 490 | 5.6 | 5.5 | 4 | 1.1 | 0.93 |
| 60 - 120 | 11560 | 462 | 5.4 | 6.9 | 5 | 1.4 | 1.1 |
|  |  |  |  |  |  |  |  |

Table S2. Results of two-way ANOVAs performed to evaluate the existence of significant differences in the bioremediation (POM, DIN-N and DIP-P concentration in outflowing water and OM present in sand filter substratum) of different IMTA designs tested in the present study using as extractive species polychaetes (*Arenicola marina* – Amar and *Hediste diversicolor* - Hdiv) and halophyte plants (*Salicornia ramosissima* - Sram) cultured in the same tank (1T) or in two separate tanks. Significant differences were considered at *p*<0,05.

| *Factor* | *F - value* | *p - value* | *R_2_ (%)* |
| --- | --- | --- | --- |
|  | | | |
| *Figure 2: POM monitored in outflowing water between different IMTA designs* | | | |
| Polychaete species | 0.04 | 0.853 | 14.28 |
| IMTA design | 0.22 | 0.647 |  |
| Polychaete species x IMTA design | 2.41 | 0.140 |  |
|  |  |  |  |
| *Figures 3a: OM monitored in 0-20 mm substratum depth between different IMTA designs* | | | |
| Polychaete species | 5.53 | 0.032 | 32.61 |
| IMTA design | 0.01 | 0.931 |  |
| Polychaete species x IMTA design | 2.25 | 0.153 |  |
|  | | | |
| *Figures 3b: OM monitored in 20-100 mm substratum depth between different IMTA designs* | | | |
| Polychaete species | 0.28 | 0.607 | 3.13 |
| IMTA design | 0.19 | 0.666 |  |
| Polychaete species x IMTA design | 0.04 | 0.853 |  |
|  |  |  |  |
| *Figure 5: DIN-N monitored in outflowing water between different IMTA designs* | | | |
| Polychaete species | 0.62 | 0.441 | 46.41 |
| IMTA design | 2.79 | 0.114 |  |
| Polychaete species x IMTA design | 12.92 | 0.002 |  |
|  |  |  |  |
| *Figure 6: DIP-P monitored in outflowing water between different IMTA designs* | | | |
| Polychaete species | 0.00 | 0.991 | 12.63 |
| IMTA design | 0.43 | 0.522 |  |
| Polychaete species x IMTA design | 1.47 | 0.243 |  |
|  |  |  |  |

Table S3. *Post-hoc* Tukey HSD tests performed to evaluate the existence of significant differences in the bioremediation (POM, DIN-N and DIP-P concentration in outflowing water and OM present in sand filter substratum) of different IMTA designs tested in the present study using as extractive species polychaetes (*Arenicola marina* – Amar and *Hediste diversicolor* - Hdiv) and halophyte plants (*Salicornia ramosissima* - Sram) cultured in the same tank (1T) or in two separate tanks. Significant differences were considered at *p*<0,05.

| Pair wise test |  | OM in 0-20 mm substratum depth | |  |  | DIN-N monitored in outflowing water | |  |
| --- | --- | --- | --- | --- | --- | --- | --- | --- |
|  |  | *T-value* | *p* |  |  | *T-value* | *p* |  |
|  |  |  |  |  |  |  |  |  |
| 2T^Amar+Sram^ – 1T ^amar+Sram^ |  | 0.78 | 0.863 |  |  | 1.87 | 0.279 |  |
| 1T ^Hdiv+Sram^ – 1T ^amar+Sram^ |  | -0.57 | 0.938 |  |  | 2.42 | 0.113 |  |
| 2T ^Hdiv+Sram^ – 1T ^amar+Sram^ |  | -1.91 | 0.261 |  |  | -0.79 | 0.856 |  |
| 1T ^Hdiv+Sram^ – 2T ^amar+Sram^ |  | -1.35 | 0.544 |  |  | 0.55 | 0.945 |  |
| 2T ^Hdiv+Sram^ – 2T ^amar+Sram^ |  | -2.69 | 0.069 |  |  | -2.66 | 0.073 |  |
| 2T ^Hdiv+Sram^ – 1T ^Hdiv+Sram^ |  | -1.34 | 0.553 |  |  | -3.21 | 0.025 |  |

Table S4. Results of two-way ANOVAs performed to evaluate the existence of significant differences in the productivity of polychaetes (*Arenicola marina* – Amar and *Hediste diversicolor* – Hdiv) and halophytes (*Salicornia ramosissima* - Sram) cultured under IMTA designs with extractive species in the same tank (1T) or in two separate tanks (2T). Significant differences were considered at *p*<0,05.

| *Factor* | *F - value* | *p - value* | *R_2_ (%)* |
| --- | --- | --- | --- |
|  | | | |
| *Table 3: Biomass of polychaetes between different IMTA designs* | | | |
| Polychaete species | 54.20 | 0.000 | 78.5 |
| IMTA design | 0.09 | 0.772 |  |
| Polychaete species x IMTA design | 4.14 | 0.059 |  |
|  |  |  |  |
| *Table 4: Density of halophyte plants between different IMTA designs* | | | |
| Polychaete species | 2.73 | 0.118 | 33.83 |
| IMTA design | 5.08 | 0.039 |  |
| Polychaete species x IMTA design | 0.36 | 0.556 |  |
|  |  |  |  |
| *Table 4: Total plant biomass of halophyte plants between different IMTA designs* | | | |
| Polychaete species | 1.59 | 0.225 | 58.66 |
| IMTA design | 21.11 | 0.000 |  |
| Polychaete species x IMTA design | 0.00 | 0.964 |  |
|  |  |  |  |
| *Table 4: Aboveground biomass of halophyte plants between different IMTA designs* | | | |
| Polychaete species | 1.39 | 0.255 | 56.56 |
| IMTA design | 19.43 | 0.000 |  |
| Polychaete species x IMTA design | 0.00 | 0.992 |  |
|  |  |  |  |
| *Table 4: Belowground biomass of halophyte plants between different IMTA designs* | | | |
| Polychaete species | 0.27 | 0.613 | 51.60 |
| IMTA design | 16.69 | 0.001 |  |
| Polychaete species x IMTA design | 0.10 | 0.751 |  |
|  |  |  |  |
| *Figure 7: Plant average weight at day 0 between different IMTA designs* | | | |
| Polychaete species | 0 | 1 | 22.04 |
| IMTA design | 4.42 | 0.052 |  |
| Polychaete species x IMTA design | 0.1 | 0.751 |  |
|  |  |  |  |
| *Figure 7: Plant average weight at day 60 between different IMTA designs* | | | |
| Polychaete species | 1.07 | 0.316 | 67.87 |
| IMTA design | 32.6 | 0.000 |  |
| Polychaete species x IMTA design | 0.12 | 0.729 |  |
|  |  |  |  |

Table S5. *Post-hoc* Tukey HSD tests performed to evaluate the existence of significant differences in the productivity of polychaetes (*Arenicola marina* – Amar and *Hediste diversicolor* – Hdiv) and halophytes (*Salicornia ramosissima* - Sram) cultured under IMTA designs with extractive species cultured in the same tank (1T) or in two separate tanks (2T). Significant differences were considered at *p*<0,05.

| Pair wise test | Total polychaetes biomass | |  | Plant density | |  | Total plant biomass | |  | Plant aboveground biomass | |  | Plant belowground biomass | |  |  | Average weight day 60 | |
| --- | --- | --- | --- | --- | --- | --- | --- | --- | --- | --- | --- | --- | --- | --- | --- | --- | --- | --- |
|  | *T-value* | *p* |  | *T-value* | *p* |  | *T-value* | *p* |  | *T-value* | *p* |  | *T-value* | *p* |  |  | *T-value* | *p* |
|  |  |  |  |  |  |  |  |  |  |  |  |  |  |  |  |  |  |  |
| 2T^Amar+Sram^ – 1T ^amar+Sram^ | 1.65 | 0.382 |  | -2.02 | 0.222 |  | -3.22 | 0.025 |  | -3.12 | 0.030 |  | -3.12 | 0.030 |  |  | -3.92 | 0.006 |
| 1T ^Hdiv+Sram^ – 1T ^amar+Sram^ | 6.64 | 0,000 |  | 0.74 | 0.878 |  | 0.93 | 0.792 |  | 0.83 | 0.841 |  | 0.14 | 0.999 |  |  | 0.26 | 0.994 |
| 2T ^Hdiv+Sram^ – 1T ^amar+Sram^ | 5.41 | 0,000 |  | -0.43 | 0.973 |  | -2.36 | 0.127 |  | -2.28 | 0.144 |  | -2.52 | 0.094 |  |  | -3.32 | 0.02 |
| 1T ^Hdiv+Sram^ – 2T ^amar+Sram^ | 5,00 | 0.001 |  | 2.76 | 0.060 |  | 4.14 | 0.004 |  | 3.95 | 0.006 |  | -3.25 | 0.023 |  |  | 4.17 | 0.004 |
| 2T ^Hdiv+Sram^ – 2T ^amar+Sram^ | 3.77 | 0.008 |  | 1.59 | 0.409 |  | 0.86 | 0.825 |  | 0.84 | 0.834 |  | 0.59 | 0.933 |  |  | 0.59 | 0.932 |
| 2T ^Hdiv+Sram^ – 1T ^Hdiv+Sram^ | -1.23 | 0.618 |  | -1.17 | 0.654 |  | -3.28 | 0.022 |  | -3.11 | 0.031 |  | -2.66 | 0.073 |  |  | -3.58 | 0.012 |

Table S6. Results of one-way ANOVA performed to evaluate the existence of significant differences in pigments concentration and chlorophyll b/chlorophyll a (Chl a/Chl b), total carotenoids /chlorophyll and zeaxanthin/carotenoids ratios exhibited by the halophytes (*Salicornia ramosissima*) cultured under different IMTA designs (cultured in the same tank with polychaetes – 1T or in two separate tanks – 2T). Significant differences were considered at *p*<0,05.

| *Factor* | *F - value* | *p* | *R_2_ (%)* |
| --- | --- | --- | --- |
|  | | | |
| *Table 6:* Pigment concentrations found in *Salicornia ramosissima*: IMTA design *vs*  initially stocked and wild plants | | | |
| 9’-*cis*-Neoxanthin | 18.25 | 0.000 | 77.39 |
| Violaxanthin | 15.10 | 0.000 | 73.89 |
| Anteraxanthin | 0.77 | 0.529 | 12.57 |
| Lutein | 130.66 | 0.000 | 96.08 |
| Zeaxanthin | 19.38 | 0.000 | 78.42 |
| Chlorophyll *b* | 23.16 | 0.000 | 81.28 |
| Chlorophyll *a* | 19.97 | 0.000 | 78.92 |
| β,β-Carotene | 16.52 | 0.000 | 75.60 |
|  | | | |
| Figure 8a: Chl b/Chl a ratio | | | |
| IMTA design vs Initially stocked and wild plants | 22.51 | 0.000 | 80.85 |
|  |  |  |  |
| Figure 8b: Total carotenoids/Chlorophyll ratio | | | |
| IMTA design vs Initially stocked and wild plants | 21.6 | 0.000 | 80.2 |
|  |  |  |  |
| Figure 8c: Zeaxanthin/Carotenoids ratio | | | |
| IMTA design vs Initially stocked and wild plants | 18.68 | 0.000 | 77.79 |
|  |  |  |  |

Table S7. *Post-hoc* Tukey HSD tests performed to evaluate the existence of significant differences in chlorophyll b/chlorophyll a (Chl a/Chl b), total carotenoids /chlorophyll and zeaxanthin/carotenoids ratios exhibited by halophytes (*Salicornia ramosissima*) cultured under different IMTA designs (cultured in the same tank with polychaetes – 1T and in two separate tanks – 2T). Significant differences were considered at *p*<0,05.

| Pair wise test | 9’-*cis*-Neoxanthin | | Violaxanthin | | Lutein | | Zeaxanthin | | Chlorophyll *b* | | Chlorophyll *a* | | β,β-Carotene | | Chl b/Chl a ratio | | Carotenoids/ Chlorophyll ratio | | Zeaxanthin/ Carotenoids ratio | |
| --- | --- | --- | --- | --- | --- | --- | --- | --- | --- | --- | --- | --- | --- | --- | --- | --- | --- | --- | --- | --- |
|  | *T-value* | *p* | *T-value* | *p* | *T-value* | *p* | *T-value* | *p* | *T-value* | *p* | *T-value* | *p* | *T-value* | *p* | *T-value* | *p* | *T-value* | *p* | *T-value* | *p* |
|  |  |  |  |  |  |  |  |  |  |  |  |  |  |  |  |  |  |  |  |  |
| 2T – 1T | 0.33 | 0.988 | 0.56 | 0.942 | -0.09 | 1.000 | -0.20 | 0.997 | -1.35 | 0.544 | -1.32 | 0.565 | -1.10 | 0.693 | -1.28 | 0.586 | 1.93 | 0.254 | 0.58 | 0.937 |
| Initial – 1T | 6.09 | 0.000 | 4.82 | 0.001 | 15.88 | 0.000 | -4.28 | 0.003 | 6.06 | 0.000 | 5.34 | 0.000 | 4.30 | 0.003 | 6.04 | 0.000 | -3.96 | 0.006 | -4.05 | 0.005 |
| Wild – 1T | 4.42 | 0.002 | 5.21 | 0.000 | 11.25 | 0.000 | -6.31 | 0.000 | 3.75 | 0.008 | 3.94 | 0.006 | 4.43 | 0.002 | 3.66 | 0.010 | -5.09 | 0.001 | -5.68 | 0.000 |
| Initial – 2T | 5.76 | 0.000 | 4.26 | 0.003 | 15.97 | 0.000 | -4.09 | 0.004 | 7.41 | 0.000 | 6.66 | 0.000 | 5.41 | 0.000 | 7.32 | 0.000 | -5.84 | 0.000 | -4.63 | 0.001 |
| Wild – 2T | 4.09 | 0.004 | 4.64 | 0.001 | 11.34 | 0.000 | -6.11 | 0.000 | 5.11 | 0.001 | 5.26 | 0.000 | 5.53 | 0.000 | 4.94 | 0.001 | -7.02 | 0.000 | -6.26 | 0.000 |
| Wild – Initial | -1.67 | 0.370 | 0.38 | 0.980 | -4.63 | 0.001 | -2.02 | 0.220 | -2.30 | 0.140 | -1.40 | 0.515 | 0.12 | 0.999 | -2.38 | 0.122 | -1.18 | 0.646 | -1.63 | 0.391 |
